# Bioenergetics of Mammalian Sperm Capacitation

**DOI:** 10.1155/2014/902953

**Published:** 2014-03-25

**Authors:** Alessandra Ferramosca, Vincenzo Zara

**Affiliations:** Dipartimento di Scienze e Tecnologie Biologiche ed Ambientali, Università del Salento, Via Provinciale Lecce-Monteroni, 73100 Lecce, Italy

## Abstract

After ejaculation, the mammalian male gamete must undergo the capacitation process, which is a prerequisite for egg fertilization. The bioenergetics of sperm capacitation is poorly understood despite its fundamental role in sustaining the biochemical and molecular events occurring during gamete activation. Glycolysis and mitochondrial oxidative phosphorylation (OXPHOS) are the two major metabolic pathways producing ATP which is the primary source of energy for spermatozoa. Since recent data suggest that spermatozoa have the ability to use different metabolic substrates, the main aim of this work is to present a broad overview of the current knowledge on the energy-producing metabolic pathways operating inside sperm mitochondria during capacitation in different mammalian species. Metabolism of glucose and of other energetic substrates, such as pyruvate, lactate, and citrate, is critically analyzed. Such knowledge, besides its obvious importance for basic science, could eventually translate into the development of novel strategies for treatment of male infertility, artificial reproduction, and sperm selection methods.

## 1. Introduction

Sperm capacitation occurs in the female genital tract and represents an essential prerequisite for oocyte fertilization [1, 2]. Overall, capacitation is a complex process and involves a series of structural and functional changes of spermatozoa including membrane modifications, modulation of enzyme activities, and protein phosphorylation. Furthermore, capacitation is accompanied by sperm hyperactivation which is characterized by an increase in flagellar bend amplitude and, usually, beat asymmetry. This change in motility helps sperm to swim through oviductal mucus and to penetrate the zona pellucida of the oocyte after acrosome reaction [2–4]. All these changes occurring during capacitation require an adequate supply of energy and, therefore, imply a fine sperm energy management.

Sperm capacitation and related processes are the result of several intracellular biochemical events, such as oxidation of energy substrates, phosphorylation of proteins involved in signal transduction through the plasma membrane, and conversion of chemical energy into mechanical energy in the axoneme. However, the metabolic pathways activated during sperm capacitation and hyperactivation, as well as the related energy sources and the molecular mechanisms of regulation, are still poorly understood.

**Figure 1:**
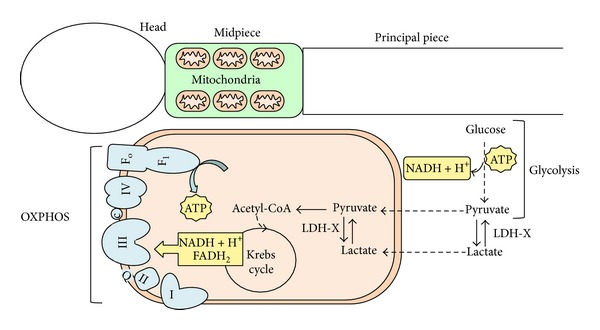
Subcellular compartimentalization of OXPHOS and glycolysis in spermatozoa. OXPHOS system is composed of five multimeric complexes. Electron transport from Complex I to Complex IV is coupled to ATP-synthesis. Reducing equivalents (NADH and FADH_2_) produced by glycolysis and Krebs cycle reactions are transferred to membrane bound electron transport chain. c, cytochrome c; Q, ubiquinone.

Glycolysis and mitochondrial oxidative phosphorylation (OXPHOS) are the two major metabolic pathways which generate cellular energy in the form of ATP. In spermatozoa, these metabolic pathways are localized in distinct cellular subcompartments. OXPHOS occurs in mitochondria, which are localized exclusively in the sperm midpiece, whereas glycolysis takes place mainly in the fibrous sheath of the flagellum where glycolytic enzymes are tightly anchored (Figure 1). Smaller quantities of glycolytic enzymes can be found in other sperm locations, like head. This could be linked with the presence of separate glucose transporters (GLUTs) in sperm areas far from flagellum [5]. Furthermore, the main substrate for glycolysis is glucose which is metabolized to pyruvate and/or lactate in anaerobic conditions. The substrates for mitochondrial OXPHOS are more diversified and are generally represented by different molecules derived from the catabolism of carbohydrate, lipid, and protein. Furthermore, glycolysis and mitochondrial OXPHOS are not alternative pathways, as often reported in the literature, yet the first pathway is preliminary to the second when the complete and aerobic oxidation of glucose is taken into account.

However, in recent years conflicting data on sperm bioenergetics have been reported [6–10]. Several authors have proposed that the cytosolic process of glycolysis represents the main source of ATP in sperm [7, 11–13]. By contrast, other researchers have emphasized the importance of sperm mitochondria in the aerobic production of energy mainly because glycolysis is characterized by a lower yield in terms of molecules of ATP produced per molecule of glucose metabolized [14–19]. Mitochondria are indeed capable of generating much more molecules of ATP after the complete oxidative breakdown of glucose via pyruvate and acetyl-CoA.

A general concept that emerged from the various studies carried out on this topic is that spermatozoa exhibit a great versatility in their metabolism. These cells are indeed able to use different substrates and to activate different energy-producing pathways in dependence of the fertilization stages and of the different environments in which they operate [20, 21]. In addition, the preferred metabolic pathway seems to be highly species specific [9].

In this review, we focus our attention on the mechanisms of energy production during mammalian sperm capacitation and hyperactivation. Both processes require an adequate supply of ATP in order to sustain all the changes occurring during this functional stage of sperm life.

## 2. Glucose and Sperm Capacitation

The addition of glucose, and not of fructose, appears necessary in order to initiate the complex series of events implied in the whiplash motility associated with fertilizing ability and in the acrosome reaction in the mouse and rat [22–24]. Sperm activation was in fact inhibited to a various degree in glucose-free medium, thereby suggesting a key role of this hexose for capacitation, motility hyperactivation, and oocyte-fusion in mammalian spermatozoa including humans [24–31]. Glucose was also required for capacitation in hamster [32] and may also be necessary for optimal capacitation in macaque sperm [33]. In contrast, glucose inhibited capacitation in guinea pig [34], bovine [35] and dog sperm, where* in vitro* capacitation is carried out in a medium without this hexose [36].

The main pathway of glucose utilization is represented by cytosolic glycolysis both in somatic and sperm cells. An important role in supplying cells with energy is played by different membrane proteins that transport hexoses through the lipidic bilayer [5]. Glucose tranporters, or GLUTs, are a family of 13 proteins that facilitate the transport of sugars and have a peculiar distribution in different tissues as well as a particular affinity for substrates. In this way, glycolytic flux, as well as the subsequent putative substrate entry to mitochondrial respiration, is firstly regulated at this point [5, 37]. In particular, in the sperm cell, the modulation of glucose transport could be achieved by the presence of various GLUTs isoforms in a peculiar and species-specific localization. Another important regulatory point of sperm glycolysis, besides the control of sugar uptake, is the subsequent hexose phosphorylation. This conversion is necessary, since monosaccharides have to be converted into glucose 6-phosphate, before being introduced into the general sperm metabolic system. Kinetics and specificity of hexose kinases are able to modulate glucose phosphorylation in sperm cells [38, 39]. The most important of these enzymes seems to be hexokinase-I, which shows a very high affinity for glucose and, at least in boar sperm [39], a much lower affinity to other monosaccharides, such as fructose, sorbitol, and mannose. Therefore, mammalian sperm seem to be able to develop a very efficient metabolic response in the presence of very low glucose concentrations, showing one of the most efficient metabolic responses among mammalian cells [37].

The principal piece of the sperm tail is devoid of mitochondria and enriched in glycolytic enzymes, such as hexokinase, phosphoglucokinase isomerase, phosphofructokinase, glyceraldehyde 3-phosphate dehydrogenase, and aldolase [40, 41]. Many of these enzymes are sperm-specific isoenzymes, with peculiar kinetic and regulatory properties, which are distinct from the isoenzymes present in somatic cells [42–46]. This enzyme localization, therefore, indicates that glycolytic ATP production occurs predominantly in the principal piece of spermatozoa. In human spermatozoa, glycolytic ATP production was suggested to be required for hyperactivated motility. In comparison to sperm from other species, human spermatozoa obtain a high proportion of their energy from glycolysis and the effect of glucose or fructose on motility can be explained by their effect on ATP levels.

Very interestingly, a novel carrier of adenine nucleotide (AAC, ADP/ATP carrier) has been identified in the sperm fibrous sheath [47]. Normally, this carrier protein is located in the inner membrane of mitochondria where it shuttles ADP against ATP through the impermeable lipidic environment of the membrane. This novel spatial location of AAC in the sperm fibrous sheath, in the vicinity of many glycolytic enzymes, suggests an ordered organization which, under certain aspects, resembles the spatial organization of the mitochondrial respiratory chain. In the sperm fibrous sheath, AAC may permit the flux of nucleotides between flagellar glycolysis, protein phosphorylation, and mechanisms of motility [47].

To understand the peculiar functional and phenotypic characteristics of sperm mitochondria in different mammalian species, the metabolism of glucose was studied [37]. The obtained results suggested that differences between mammalian species could be linked to changes in sperm energy management. By using a metabolomic approach, two distinctive sperm phenotypes were analyzed: the “boar spermatozoa,” which are characterized by low average motility and short survival capacity inside the female vaginal tract, and the “dog spermatozoa” with fast average motility and long survival capacity inside the female vaginal tract. The results obtained from this study suggested that glycolysis plays a significant role as an energy source in boar sperm, while the dog sperm phenotype is characterized by an active anabolic metabolism and, probably, an active pentose phosphate cycle pathway [48].

## 3. Mitochondrial OXPHOS and Sperm Capacitation

Several studies demonstrated that mitochondrial respiration increases abruptly during capacitation in mice sperm [49, 50]. This stimulation of mitochondrial respiration is probably due to the availability of oxidizable substrates, which are usually present in the capacitation medium. Results obtained in human sperm [51, 52] showed that* in vitro* capacitation was concomitant with a peak in O_2_ consumption. On the other hand, capacitated boar sperm did not show a significant increase of O_2_ consumptions when compared with freshly obtained cells [53].

A methodology originally developed for animal studies [54–56] has recently been used also in human sperm in order to better understand the changes in mitochondrial energetic metabolism occurring in distinct stages of spermatozoa life. This technique consists in the measure of mitochondrial oxygen consumption, which is a quantitative measure of mitochondrial respiration efficiency, in demembranated sperm cells by hypotonic swelling. In these experimental conditions, human sperm mitochondrial respiration can be assayed in a highly reproducible way in the presence of different respiratory substrates [19, 57, 58]. However, it must be underlined that demembrenated cells lack all of the regulatory mechanisms linked to the first steps of the glycolytic pathway, as well as the regulatory mechanisms dealing with the incorporation of the exogenous substrates to mitochondrial respiration.

When oxygen consumption was assayed in swim-up selected spermatozoa, incubated under capacitating conditions after hypotonic swelling, an impressive increase in mitochondrial respiration capacity was found [52]. The high mitochondrial respiratory efficiency, which was about 20 times higher than that measured in basal samples, remained stable up to 24 h after the swim-up treatment. This increase in mitochondrial respiration capacity, measured under capacitating conditions, seems finalized to sustain the hyperactivated motility observed in about 90% of swim-up selected sperm. OXPHOS is indeed much more efficient, in terms of energy yield, than cytosolic glycolysis, thereby supplying sperm cells with higher amounts of ATP. It is also important to underline that the physiology of sperm mitochondria was preserved after the swim-up treatment and during capacitation, since the respiratory control ratio (a useful parameter for evaluation of OXPHOS efficiency), the substrate specificity and the inhibitor sensitivity were similar to those of basal samples [52].

Morphological changes of human sperm mitochondria were also observed during capacitation using X-ray microscopy [59]. After sperm incubation under capacitating conditions, a remarkable change in mitochondria towards a more loosely wrapped morphology, possibly resulting from an increase in mitochondrial volume, was observed. This is in line with the general concept that mitochondria are dynamic organelles which can change their size and shape depending on their metabolic status.

Mitochondria of sperm and somatic cells, in addition to their basic role in oxidative energy generation, are also a source of reactive oxygen species (ROS). These organelles are indeed able to convert 0.2–2% of the oxygen taken up by the cells to ROS, which, at low concentrations, play a physiological role in many cellular processes [60, 61]. In particular, it has been demonstrated that coincubation of spermatozoa with small amounts of hydrogen peroxide stimulates sperm capacitation, hyperactivation, and acrosome reaction [62]. However, the molecular mechanisms by which ROS exert these effects in mammalian spermatozoa are still largely unknown.

What is the relationship between high mitochondrial respiratory efficiency, ROS, and biochemical modifications observed during sperm capacitation? An increase in the mitochondrial respiration rate could enhance the production of ROS, which seems to partecipate in molecular events implicated in sperm capacitation and hyperactivation. On the other hand, sperm mitochondria could become a target of elevated levels of ROS and, if this happens, the process of OXPHOS can be severely affected as a consequence of protein and lipid damage [58]. This aspect appears particularly intriguing and appropriate experiments should be designed to investigate the role of mitochondria as a source, and not as a target, of ROS during sperm capacitation. Interestingly, mitochondrial respiratory control ratio calculated in human sperm during capacitation suggested a good coupling between respiration and phosphorylation and, thus, a preserved integrity of mitochondria during capacitation and hyperactivated sperm motility [52].

Moreover, a recent study carried out in boar sperm suggested that mitochondrial control of processes such as motility and capacitation could be linked to other processes than the mere ATP production [63]. In fact, the specific oligomycin inhibition of mitochondrial ATP synthase reduced sperm motility and the achievement of* in vitro* capacitation, without modifying the overall intracellular ATP levels, the rhythm of O_2_ consumption and the activity of the mitochondrial respiratory chain. In addition, these results seem to indicate that boar sperm mitochondria are mainly in an uncoupled status, reaching coupling during the induction of acrosome exocytosis. Therefore, the mitochondrial ROS production could be a modulator mechanism by which sperm acquire fertilizing ability during their life.

## 4. Role of Pyruvate and Lactate in Sperm Capacitation: A Molecular Link between Cytosolic Glycolysis and Mitochondrial OXPHOS

**Figure 2:**
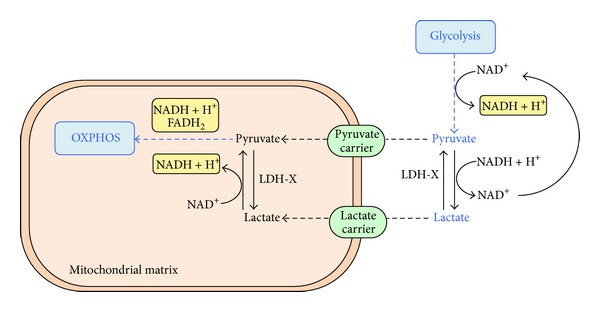
Sperm metabolism of pyruvate and lactate. Pyruvate is a glycolitc product. Lactate derives from the reduction of pyruvate, especially in anaerobic conditions, and this reaction regenerates the cytosolic NAD^+^ necessary for the progress of glycolysis. Pyruvate and lactate produced in the cytosol of sperm cells can then be transported inside mitochondria for further metabolization.

Pyruvate and lactate are present at high concentration, along with glucose, in oviductal fluid [20] and, hence, they are commonly used as energy substrates by mammalian spermatozoa. Furthermore, the metabolism of pyruvate is strictly correlated to that of lactate, which derives from the enzymatic reduction of pyruvate by lactate dehydrogenase (Figure 2). This reaction, especially in anaerobic conditions, regenerates cytosolic NAD^+^ necessary for the progress of glycolysis. Cytosolic lactate can also be transported inside mitochondria of sperm cells by the mitochondrial lactate carrier for further metabolization [9, 64]. Sperm mitochondria, because of the presence of a novel isoenzymatic form of lactate dehydrogenase (LDH-X or LDH-C4) [65, 66], specific for sperm mitochondria, are also able to reoxidize lactate to pyruvate in the mitochondrial matrix. Therefore, LDH-X is present both in the mitochondrial matrix and in the cytosol of spermatozoa, with a net prevalence in the cytosol [67]. The joined operation of the cytosolic and mitochondrial LDH and of the lactate carrier allows, on one hand, the progress of glycolysis by the production of NAD^+^ and, on the other, the progress of OXPHOS, by the transport of reducing equivalents from the cytosol into mitochondria [9, 68].

It has been recently demonstrated that pyruvate accelerates glycolysis and promotes capacitation in human spermatozoa [31]. Hereng and colleagues suggested that exogenous pyruvate is able to accelerate glycolysis through the LDH-mediated conversion of pyruvate to lactate and the concomitant regeneration of cytosolic NAD^+^. In particular, authors found that, in the absence of metabolic substrates, endogenous ATP concentrations were reduced in the presence of inhibitors of mitochondrial respiration, such as NaCN, rotenone, and antimycin A. ATP levels were not restored when 5 mM of glucose, lactate, or pyruvate were added separately. However, when a combination of glucose and pyruvate was added, ATP levels were similar to those seen in noninhibited control cells. Interestingly, a combination of glucose and lactate did not reestablish the ATP levels under the same conditions. These findings indicate that exogenous pyruvate enhances ATP production because of its ability to regenerate NAD^+^ following its conversion to lactate and, therefore, independently of mitochondrial respiration [31]. In this context, it is useful to remind that the complete mitochondrial oxidation of pyruvate in bovine, rabbit, rat, mouse and human spermatozoa requires the simultaneous addition of malate. It has been proposed that this latter molecule has a stimulatory role on pyruvate oxidation by sperm mitochondria [56, 57, 69–71].

## 5. The Role of Citrate in Sperm Capacitation

It has been found that boar sperm efficiently metabolize citrate and lactate through a metabolic pathway regulated by LDH [72]. In particular, the incubation of boar sperm in the presence of citrate induced the appearance of extracellular lactate, which was subsequently uptaken by sperm in order to be metabolized through mitochondrial respiration. This apparent nonsense process would yield significant amounts of NADH that would be needed for the cell to maintain its function.

Reducing equivalents, in the form of NADPH, are produced in the pentose phosphate cycle pathway. This pathway provides means by which glucose can be oxidized to generate NADPH, which appears to participate in supporting a precise onset of tyrosine phosphorylation in the sperm flagellum leading to hyperactivated motility in capacitated spermatozoa [73] from several mammalian species. In particular, NADPH is essential for the activity of an NADPH-oxidase which produces radical species promoting sperm function and tyrosine phosphorylation [74, 75], although ROS may also be produced by the mitochondrial electron transport chain [76]. However, the observation that NADPH is detectable also in the absence of glucose suggested the existence of other pathways providing NADPH in spermatozoa [27, 28]. The existence of another pathway which contributes to NADPH production was proposed in a recent study [77], where a pivotal role of the mitochondrial citrate carrier (CIC) in sperm capacitation was demonstrated. CIC is an integral protein of the inner mitochondrial membrane that catalyzes the transport of citrate from mitochondria, where this molecule is formed in the first reaction of the Krebs cycle, to cytosol. CIC, therefore, plays a central role in intermediary metabolism, because the citrate efflux from mitochondria provides the carbon source and NADPH for several metabolic processes, such as* de novo* fatty acid synthesis, sterol biosynthesis, insulin secretion, and histone acetylation [78].

Cappello and colleagues [77] demonstrated that CIC was present in the midpiece of human ejaculated spermatozoa, providing evidence for a physiological role of this protein in capacitation, acrosome reaction, and sperm metabolism. In particular, in order to evaluate whether CIC and its substrate citrate promote capacitation, authors considered two representative parameters of capacitation: cholesterol efflux from the plasma membrane, which modifies sperm plasma membrane fluidity [79–81] and protein tyrosine phosphorylation. Interestingly, they found that the inhibition of CIC resulted in the reduction of these molecular events. It was also demonstrated that both citrate and glucose promoted capacitation most probably by a common mechanism consisting in the stimulation of insulin secretion by sperm cells. Mitochondrial CIC, therefore, represents a new factor involved in the molecular events underlying sperm capacitation process.

## 6. Concluding Remarks

Sperm capacitation is a complex process which involves profound structural and functional changes in male gamete thereby preparing it for acrosome reaction and egg fertilization. These changes are only partially known at a molecular level but most of them require an adequate supply of energy. For this reason, spermatozoa modify their metabolism in order to adequately sustain capacitation and the closely related phenomen of sperm hyperactivation. Although several studies have been carried out on sperm metabolism during capacitation, the overall biological and biochemical framework emerging from these investigations is still unclear.

First, it is currently unknown what the main destiny of the extra-energy required during capacitation is. Indeed, it can be used, to a various degree, for sperm hyperactivation and, therefore, for gamete motility, for protein phosphorylation, for sperm structural changes, for maintaining adequate concentrations of intracellular ions, and in this respect mainly Ca^++^ ions, and eventually for the synthesis of new molecules. Furthermore, it is also unclear the priority, in terms of importance and of sequence of events, among all these energy-demanding reactions. Therefore, future work can be addressed to this direction in order to unveil this complex network of events guiding sperm capacitation and then egg fertilization.

Second, the metabolic pathway which is mainly required for the production of this extra-energy during capacitation and sperm hyperactivation is also a matter of investigation. For decades, researchers have been debating whether sperm cells get the necessary energy from glycolysis or from mitochondrial OXPHOS. Some studies reported that glucose is beneficial to sperm for optimal capacitation, although it is still unclear whether glucose is required for providing extra metabolic energy through glycolysis or for generating other metabolic products. Energy generated by glycolysis could play a role in capacitation by sustaining sperm hyperactivation, thereby ensuring a good fertilizing potential over time. On the other hand, sperm capacitation can also depend on the increase in mitochondrial oxidative metabolism. It appears, therefore, that the metabolic source of ATP, that is, glycolysis or mitochondrial OXPHOS, may change in different mammalian species and may also depend, in the same species like humans, on the substrates available and/or on the oxygen concentration. In other words, spermatozoa respond to changes in energy demand by adjusting glycolytic flux or mitochondrial respiration in relation to the metabolic status of the cell.

Third, the metabolic pathways activated during sperm capacitation may serve other purposes, such as the synthesis of ROS which, at low levels, are required to promote capacitation. The respiratory chain of sperm mitochondria, under physiological conditions, is indeed capable of producing low levels of ROS. Furthermore, cytosolic glucose and mitochondrial citrate may generate, via distinct metabolic pathways, NADPH which is also important to promote sperm capacitation. Very intriguing is the role of the sperm mitochondrial CIC which, in addition to NADPH production, is also involved in the insulin secretion by male gamete.

The overall picture emerging from all these investigations is rather complex but, at the same time, very challenging. In fact, the recent discovery of new unexpected molecules involved in regulation of sperm metabolism and in promotion of sperm capacitation, could open up new avenues of investigation in the study of physiology and pathology of male reproduction.
